# Hollow Fiber Microreactor Combined with Digital Twin to Optimize the Antimicrobial Evaluation Process

**DOI:** 10.3390/mi15121517

**Published:** 2024-12-20

**Authors:** Kazuhiro Noda, Toshihiro Kasama, Marie Shinohara, Masakaze Hamada, Yukiko T. Matsunaga, Madoka Takai, Yoshikazu Ishii, Ryo Miyake

**Affiliations:** 1 Bioengineering, School of Engineering, The University of Tokyo, Tokyo 113-8656, Japan; kasama@g.ecc.u-tokyo.ac.jp (T.K.); takai@bis.t.u-tokyo.ac.jp (M.T.); trmiyake@mail.ecc.u-tokyo.ac.jp (R.M.); 2 Institute of Industrial Science, The University of Tokyo, Tokyo 153-8505, Japan; marie-s@g.ecc.u-tokyo.ac.jp (M.S.); matsunaga-iis@g.ecc.u-tokyo.ac.jp (Y.T.M.); 3 Department of Microbiology and Infectious Diseases, Toho University School of Medicine, Tokyo 143-8540, Japan; masakaze-hamada@g.ecc.u-tokyo.ac.jp (M.H.); ishiiyo@hiroshima-u.ac.jp (Y.I.)

**Keywords:** antimicrobial resistance, fluidics, miniaturization, numerical simulation

## Abstract

In order to reproduce pharmacokinetics (PK) profiles seen in vivo, the Hollow Fiber Infection Model (HFIM) is a useful in vitro module in the evaluation of antimicrobial resistance. In order to reduce the consumption of culture medium and drugs, we developed a hollow fiber microreactor applicable to the HFIM by integrating the HFIM function. Next, we constructed a novel control method by using the “digital twin” of the microreactor to achieve precise concentration control. By integrating functions of the HFIM, the extra-capillary space volume was reduced to less than 1/10 of conventional HFIM. The control method with the digital twin can keep drug concentration in the extra-capillary space within an error of 10% under simulated drug destruction. The control method with the digital twin can also stabilize the drug concentration both in the intra-capillary space and the extra-capillary space within 15 min.

## 1. Introduction

Antimicrobial resistance (AMR), which makes the drugs used in the treatment of bacteria ineffective, has become a major threat to global health [1,2]. In 2019, 4.95 million (3.62–6.57) deaths were associated with bacterial AMR [3]. In order to overcome the AMR threat, it is crucial to develop novel antimicrobial drugs [4]. In order to reproduce pharmacokinetics (PK) profiles seen in vivo, the Hollow Fiber Infection Model (HFIM), which is an in vitro module, is often used in the evaluation of antimicrobial resistance [5,6,7,8,9].

**Figure 1:**
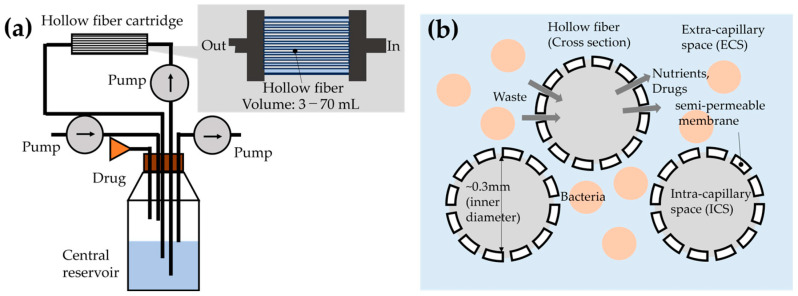
(**a**) Schematic diagram of HFIM, which has three pumps and one hollow fiber cartridge. Bacteria are cultured in the closed space in the hollow fiber cartridge. (**b**) Cross-sectional image of hollow fiber cartridge and material exchanges through hollow fibers.

Figure 1a shows a schematic diagram of the HFIM [10,11], which uses a hollow fiber cartridge, several reservoirs, and pumps. The hollow fiber cartridge is attached to a central reservoir that includes a culture medium and drugs. The pump in the circuit supplies the medium and the drugs to the hollow fiber cartridge where bacteria are cultured. Fresh medium is continuously supplied to the central reservoir with a fixed flow rate. The liquid in the central reservoir is removed by a pump, and the liquid volume is kept constant. The HFIM is a biologically safe system in that bacteria are cultured in an enclosed space in the hollow fiber cartridge.

A cross-sectional image of the hollow fiber cartridge is shown in Figure 1b. Semi-permeable membranes separate intra-capillary space (ICS) and extra-capillary space (ECS). Only the ICS is connected to the central reservoir where the medium and the drugs are supplied. By diffusion through the fibers, the medium and the drugs are supplied to the ECS, where bacteria are cultured. In the HFIM, bacteria are continuously cultured by supplying fresh medium, and PK profiles are reproduced by adjusting the drug concentration in the central reservoir.

Although the HFIM is a useful tool, it also has several issues that need to be solved [12]. This paper focuses on the following two issues. The first is that the HFIM requires a large amount of culture medium and drugs (Typically, ~10 L). This enlarges an environmental impact and makes it difficult to conduct many experiments in parallel. The second is that the drug concentration in the ECS cannot be ensured because the concentration is adjusted by diffusion through hollow fibers [13]. The possible reasons for the difference in the concentration of the ECS from the target value may be insufficient diffusion through hollow fibers or the destruction of the drugs by bacteria.

In this study, we developed a hollow fiber microreactor applicable to the HFIM by integrating HFIM functions to reduce the consumption of culture medium and drugs. In miniaturizing a bioreactor, high-density bacteria culturing is required, which makes the influences of bacteria metabolism on the surrounding environment more remarkable. Especially in the HFIM, drug destruction by bacteria, which changes the drug concentration in the ECS, needs to be considered. In order to control the drug concentration in the ECS accurately, we also constructed novel controlling methods for the microreactor.

In establishing the controlling method, the microreactor’s properties on drug diffusion should be estimated in advance. In order to estimate such properties and optimize the microreactor’s operation, utilization of a numerical model that represents the microreactor is an effective way [14]. In this paper, we constructed a controlling method that uses the “digital twin” of the microreactor to control the drug concentration in the ECS accurately [15,16]. We combined the numerical model that represents the microreactor with the concentration value measured in the real-world system to achieve accurate concentration control. There are several previous studies of numerical analysis in material diffusion through hollow fibers [17,18] and stable control of material concentration in hollow fiber bioreactors using advection analysis [19]. In the previous studies, drug diffusion through the hollow fibers was determined in advance, and drug destruction by bacteria was not considered. We constructed a numerical model that considers drug destruction by bacteria and a digital twin that can estimate drug diffusion through hollow fibers and accurately control the drug concentration.

In applying the digital twin concept to the microreactor, the validation of interaction between the real-world flow system and the numerical model is the key point. In order to check this point, we used sodium chloride to mimic drug administration in the microreactor. By assuring that the diffusion of sodium chloride through hollow fibers can be estimated and the concentration of sodium chloride in the ECS can be accurately controlled, we checked the validity of the developed digital twin. The validations shown above enable the digital twin to be utilized in the biological HFIM.

## 2. Materials and Methods

### 2.1. Overall System

**Figure 2:**
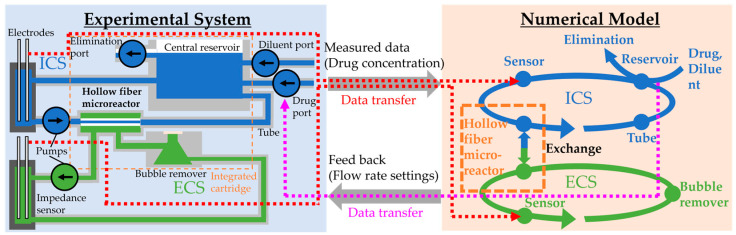
The concept of a digital twin of hollow fiber microreactor applicable to the HFIM developed in this paper. By using a combination of experimental systems and numerical models, concentrations are precisely controlled. The numerical model was calculated on PC. Details of components shown in this figure are described in Figure 3, Figure 4 and Figure 5.

The overall system design is shown in Figure 2. We constructed a hollow fiber microreactor applicable to the HFIM, including impedance sensors and pumps. The details of the hollow fiber microreactor and the impedance sensor are described in Section 2.2 and Section 2.3, respectively.

The digital twin controlled the microreactor. In the digital twin, we used a numerical model that calculates advection in flow paths and material exchange by diffusion through hollow fibers. The details of the numerical model are described in Section 2.4. In connecting the real-world microreactor and the numerical model, we used the concentration data measured in the microreactor. We used a data assimilation method with the ensemble Kalman filter to combine measured data and the results of the numerical model (Section 2.5). As feedback to the microreactor, the numerical model calculates appropriate flow rate settings to keep the concentration in the ECS at the target value (Section 2.6).

In order to check the robustness of the concentration control by the numerical model under drug destruction by bacteria, we simulated the drug destruction by bacteria in the ECS by inlet and outlet pumps (Section 2.7).

### 2.2. Construction of Hollow Fiber Microreactor

**Figure 3:**
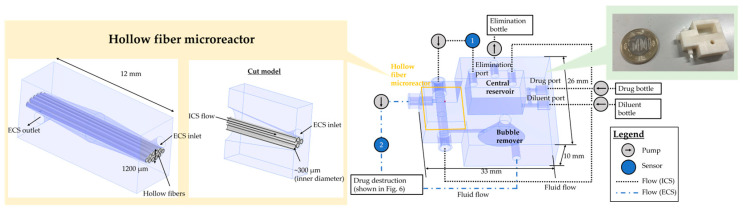
The flow system of hollow fiber microreactor applicable to the HFIM. The 3D model shows the hollow fiber microreactor created by a 3D printer. The ECS volume is less than 1/10 of conventional HFIM.

Figure 3 shows the flow system of the hollow fiber microreactor. In constructing the microreactor, we used a 3D printer (ANYCUBIC Photon M3 Plus: Shenzhen Anycubic Technology, Shenzhen, China). The 3D printer uses a UV light source and liquid crystal display to harden resin [20,21]. We used an allergy-free resin (W-64: Okamoto Chemical Industry, Saitama, Japan) to enable culturing bacteria in the printed device. Liquids were pumped into the microreactor by rotating disposable pump cassettes (MTIC1: Icomes Lab, Iwate, Japan) with stepping motors, which are controlled by a microcomputer (Raspberry Pi).

The microreactor integrates the hollow fiber cartridge and the central reservoir shown in Figure 1 and adds an air bubble remover. In the hollow fiber microreactor, 10 hollow fibers with a length of 20 mm are fixed with an adhesive to separate the ICS and the ECS. The central reservoir contains five ports: drug, diluent, and elimination ports and the other two ports are connected to the integrated cartridge. The elimination port at the central reservoir is located higher than other ports, and the liquid in the central reservoir was kept at a constant volume by eliminating liquids that overflowed from the elimination port. The bubble remover contains a flow path with the shape of an elliptical cone, which can remove air bubbles. Air bubbles can be drained from the top hole by attaching a filter that allows air to pass through. The whole ECS volume is 180 μL, which is less than 1/10 of conventional hollow fiber cartridges [22]. By reducing the size, the consumption of culture medium and drugs in the HFIM can be reduced.

Focusing on the ICS, the drug and the diluent are supplied by the two pumps and mixed in the central reservoir. The pump eliminates the solution in the reservoir when the liquid surface reaches the hole at the elimination port. The central reservoir is also connected to the hollow fiber microreactor by a circulating pump via a sensor that monitors the concentration in the ICS.

Liquids in the ECS were circulated with a pump (150.0 μL/min), and the concentration was also monitored. There are two additional pumps in the ECS to mimic the drug destruction by bacteria. The destruction was simulated by simultaneously eliminating the liquid in the ECS and supplying the diluent to the ECS with the same flow rate. Details are described in Section 2.7.

### 2.3. Sensor for Concentration Measurement

In reducing the volume of the microreactor, the size of the sensor for concentration monitoring also must be reduced. We constructed a compact sensor that can be inserted into the flow path of the microreactor.

We used sodium chloride (Special Grade: Hayashi Pure Chemical, Osaka, Japan) as a substance to mimic the drug. The concentration of sodium chloride in the flow channel can be measured by impedance [23]. By using impedance, the structure of the sensor can be simple; that is, only two electrodes are needed. We used Pt-coated titanium electrodes with a diameter of 1 mm (ND-001: TANAH PROCESS, Osaka, Japan) and measured the impedance with an impedance spectroscope (HF2IS: Zurich Instruments, Zurich, Switzerland). The voltage between a couple of electrodes was set to 10 mV, and the concentration was determined by the absolute value of the impedance at 200 kHz [24].

**Figure 4:**
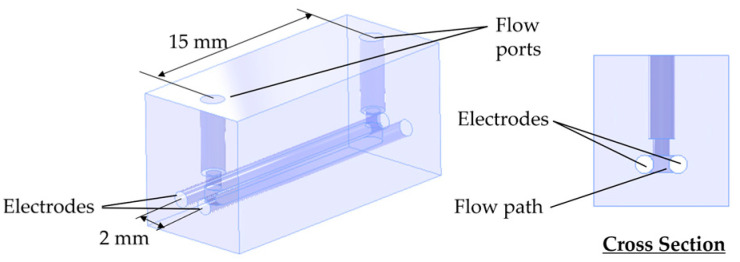
Impedance measurement device for concentration determination. Two electrodes were inserted into two holes and were connected to the impedance spectroscope.

We created a flow cell for impedance measurement to monitor the concentration in the ICS and ECS in real time. The 3D printer used to create the hollow fiber microreactor was used to create a flow path to insert the electrodes (Figure 4). The distance between the electrodes was set to 2 mm. The sensor had sensitivity in the sodium chloride concentration range used in this research (10~10 mM). Two flow ports were equipped to insert the sensor into the flow path in the microreactor.

### 2.4. Numerical Model

**Figure 5:**
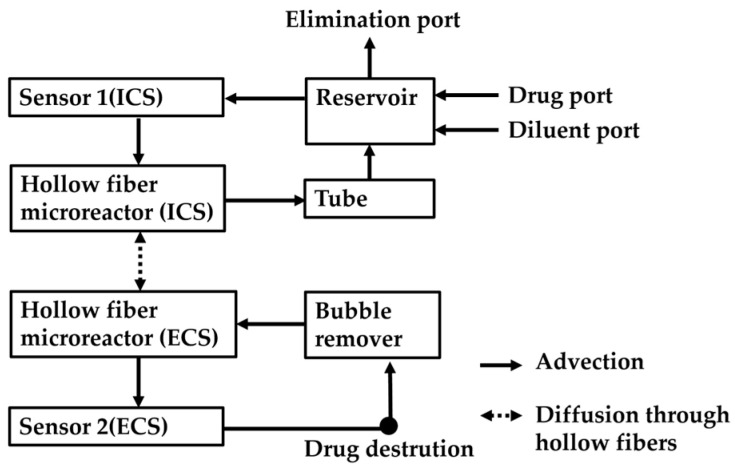
Numerical model to calculate drug advection and diffusion through hollow fibers. Advection and diffusion in the flow system shown in Figure 3 were calculated.

In this research, we constructed a numerical model that calculates drug advection and diffusion through hollow fibers. The flow system shown in Figure 3 can be represented by seven components with five inlets or outlets (Figure 5). Drug advection can be calculated by

(1) $$V_{i}\frac{{dc}_{i}}{dt}=\sum c_{in}Q_{in}-c_{i}\sum Q_{out},$$

where $c_{i}$ is the concentration in the component, $V_{i}$ is the volume of the component, and Q is the flow rates into/out of the component. Advection is represented with solid arrows in Figure 5. Drug diffusion through hollow fibers can be calculated by [25]

(2) $$V_{i}\frac{{dc}_{i}}{dt}=q(c_{ICS}-c_{ECS}),$$

where q corresponds to the flux of drug diffusion through hollow fibers, which has the same dimensions as the flow rate. The value of q should depend on the drug; hence, this value can be automatically fixed with data assimilation in this research. The whole analysis in Figure 5 is calculated using the Simulink^®^ (ver 10.6: MathWorks, Natick, MA, USA) software in this research.

### 2.5. Data Assimilation with Ensemble Kalman Filter

We used the ensemble Kalman filter (EnKF) to combine the numerical model in Figure 5 with concentration measurements [26,27]. In order to estimate the unknown value q, which corresponds to the flux through hollow fibers, we defined a state vector and observation vector in EnKF as follows:

(3) $$x=(c_{1},\ldots ,\;c_{i},\ldots ,\;c_{7},\;q),$$

(4) $$y=(c_{sensor1},c_{sensor2}),$$

where $c_{i}$ is the concentration in the component. In EnKF, an ensemble of the vector x is used to represent the distribution of state. Hereafter, the suffix j represents the j-th member of the ensemble.

The time development of $x^{j}$ was calculated as follows:

(5) $$x_{t}^{j}=F\left(x_{t-dt}^{j}\right)+w^{j},$$

where function F means the calculation of time development with the numerical model shown in Figure 5 and $w^{j}\;$is the Gaussian noise.

Each state vector was updated with measurements. The update process was calculated as follows,

(6) $$x_{t}^{a,j}=x_{t}^{j}+K_{t}\;(y_{t}+v^{j}+H_{t}x_{t}^{j})\;,$$

(7) $$K_{t}=C_{t}H_{t}^{T}{\left(H_{t}C_{t}H_{t}^{T}+R_{t}\right)}^{-1},$$

where $C_{t}$ is the covariance matrix of $x_{t}^{j},\;v^{j}$ is the Gaussian noise, $K_{t}$ is the Kalman gain, $R_{t}$ is the covariance matrix of $v^{j}$, and $H_{t}$ is the observation matrix.

In this research, concentration measurement was performed every one minute. After each measurement, state vectors were updated with measured concentration values. Since the state vectors include the value of q, which corresponds to the flux of drug diffusion through hollow fibers, drug diffusion properties through hollow fibers were automatically estimated.

### 2.6. Concentration Control with Digital Twin

Feedback controls on flow rate are required to achieve accurate drug concentration. In estimating appropriate flow rate settings, the drug destruction rate in the ECS must be estimated. We constructed a feedback control method with the following procedures:

1. Update the state vectors with EnKF [Equations (5)–(7)].
2. Assume the current state by averaging the state vectors.
3. Estimate the drug destruction rate in the ECS by the time history of concentration in the ICS and ECS.
4. Calculate the appropriate flow rate of the drug port with estimated drug destruction.

Because drug destruction in the ECS was not known in the operation of HFIM, the drug destruction rate was estimated from the measured concentration values of the two sensors from the start of the operation to the current time. The time history of the concentration in the microreactor was calculated using the numerical model with different destruction rates, and we used the Gauss–Newton method to determine the destruction rate, which minimizes the difference from the measured value [28].

### 2.7. Flow Control to Simulate Drug Destruction by Bacteria

**Figure 6:**
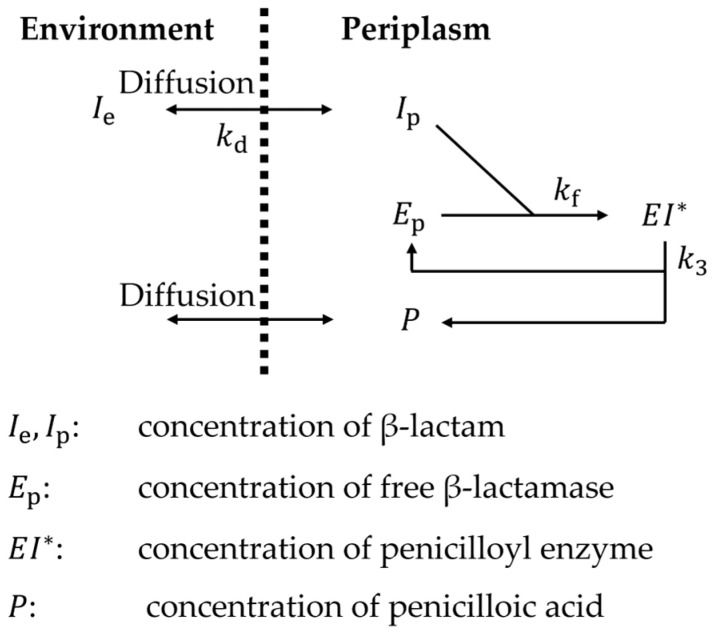
The numerical model of β-lactam destruction by *E. coli* proposed in Frére (1988) [29]. The degradation proceeds in the periplasm of *E. coli* in this model.

We applied a numerical model in Frére (1988), which considers destruction by *E. coli* [29]. They calculated the destruction of the β-lactam family, which is an antibiotic against *E. coli* [30,31]. Under steady state (Figure 6), the destruction can be calculated by

(8) $$\frac{dI_{p}}{dt}=k_{d}\left(I_{e}-I_{p}\right)-k_{f}E_{p}I_{p},$$

(9) $$\frac{dE_{p}}{dt}=k_{3}EI^{*}-k_{f}E_{p}I_{p}\;,$$

(10) $$\frac{dEI^{*}}{dt}={-k}_{3}EI^{*}+k_{f}E_{p}I_{p}\;,$$

where $I_{e}$ and $I_{p}$ are the external and periplasmic concentrations of β-lactam, $E_{p}$ is the periplasmic concentration of the enzyme, ($EI^{*}$) is the concentration of the penicilloyl enzyme, $k_{d}$ is the rate constant for β-lactam diffusion through the outer membrane, $k_{f}$ is the rate constant for the formation of penicilloyl enzyme, and $k_{3}$ is the rate constant for the penicilloyl enzyme’s hydrolysis. We used values in Table 1, which are also used in Frére (1988) [29]. The $k_{d}(I_{e}-I_{p})$ term is the destruction of penicillin in the environment. When $I_{e}=100\;\mu M$, the destruction rate is $k_{d}(I_{e}-I_{p})=0.47\;\mu M/s$. We adopted this destruction rate.

We simulated this destruction by supplying diluent to the ECS and eliminating liquid from the ECS simultaneously. We assumed an *E. coli* culture with 100 g dcw/L density [32], which is equivalent to 23% *v*/*v* for a cell size of 1 μm^3^ [33]. Considering that the volume of periplasmic space is approximately 30% of the whole cell volume [34], a 0.47 μM/s destruction rate in periplasm corresponds to 0.043 μM/s = 2.6 μM/min destruction in the free ECS volume. From the above estimation, we set the flow rate of pumps for supplying diluent to the ECS and eliminating liquid from the ECS to 2.6 μM/min × (ECS volume)/(ECS concentration).

## 3. Results and Discussion

### 3.1. Estimation of Diffusion Through Hollow Fibers in Drug Administration Process

In order to simulate the drug administration process, we introduced 10 mM sodium chloride from the drug port to the microreactor, which was filled with pure water and did not initially include sodium chloride. We set the flow rate of each port to the values shown in Table 2.

The ratio of the flow rate in the diluent and the drug port at ICS was set to enhance the sodium chloride concentration in the microreactor to 1 mM within 30 min. By diluting the 10 mM sodium chloride with pure water supplied from the dilution port, the target concentration of 1 mM was obtained. In order to enhance the sodium chloride concentration in the microreactor to 1 mM within 30 min, an additional volume of 10 mM sodium chloride was supplied from the drug port. We set the additional volume of 10 mM sodium chloride to 1/10 of the total volume of the microreactor.

**Figure 7:**
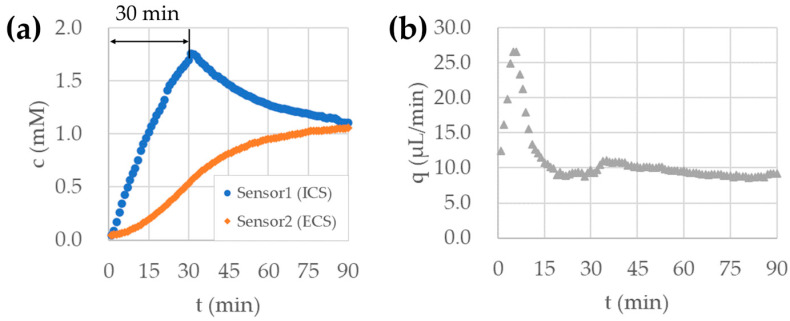
(**a**) Concentration during drug administration process in hollow fiber microreactor measured by two sensors inserted into ICS and ECS, (**b**) Flux of sodium chloride diffusion through hollow fibers estimated by EnKF.

Figure 7 shows the measured concentrations and estimated flux of sodium chloride through hollow fibers. As shown in Figure 7a, the measured concentrations in the ICS and ECS converged at 1 mM, which was the target concentration of this experiment. The concentration in the ICS initially exceeded the target concentration and then converged to the target value. This trend was caused by sodium chloride diffusion through hollow fibers. The smaller the sodium chloride diffusion is, the longer time is required for the ICS and ECS concentrations to converge to the target value.

Figure 7b shows the flux of sodium chloride through hollow fibers (the value of q), which is estimated by EnKF. After each concentration measurement was performed every minute, the ensemble of state vectors, which was written by Equation (3), was updated. In this update process, the value of q was automatically adjusted by measured concentration values. The value of q converged to around 9.0 μL/min within 30 min. This convergence was quick enough in HFIM operation, which takes several hours or days.

From the results shown above, we can conclude that the drug diffusion through hollow fibers was able to be checked using EnKF. This helps ensure the drug administration to the ECS, which cannot be achieved in conventional HFIM.

### 3.2. Validation of Concentration Control Under Drug Destruction

In simulating the drug destruction by bacteria in the ECS, we used the flow rate setting shown in Table 3. The flow rate in the elimination port and the dilution port in the ECS was determined by 2.6 μM/min × (ECS volume)/(ECS concentration). The concentration measured by the sensor equipped in the ECS (Sensor 2) was used in the ECS concentration term.

In this experiment, we set the target concentration of the ECS to 100 μM. The microreactor was initially filled with 100 μM sodium chloride. An amount of 1 mM sodium chloride was introduced from the drug port in the ICS. By diluting 1 mM of sodium chloride with pure water supplied from the dilution port in the ICS, 100 μM sodium chloride was supplied to the ICS continuously.

**Figure 8:**
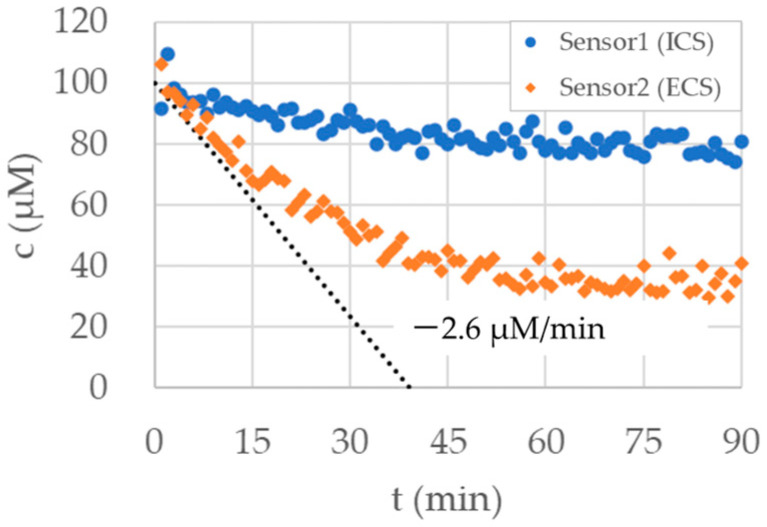
Concentration in the hollow fiber microreactor measured by two sensors inserted into ICS and ECS. The drug destruction rate in the ECS was set to −2.6 μM/min, which is represented with the dotted line. The flow rate of sodium chloride solution supplied from the drug port in the ICS was set to a constant value of 5.5 μL/min.

Figure 8 shows the measured concentrations in the ICS and the ECS with drug destruction in the ECS. The flow rate of sodium chloride solution supplied from the drug port in the ICS was set to a constant value of 5.5 μL/min, which corresponds to a continuous supplement of 100 μM sodium chloride. Despite the continuous supplement of 100 μM sodium chloride, concentrations in both the ICS and the ECS decreased due to drug destruction.

During the first 15 min, the concentration in the ECS decreased with a constant rate of −2.6 μM/min, which is represented by the dotted line in Figure 8. This trend indicates that flow control in this research that simulates drug destruction worked successfully. Due to the sodium chloride supplied by the ICS, the concentration in the ECS converged to a constant value of around 35 μM. The sodium chloride supplied from the ICS and destructed in the ECS was balanced in this convergence. The final concentration in the ECS is less than half of the target concentration, which indicates that the concentration in the ECS may not be correctly controlled by continuous supplement of solutions with the target concentration.

We compared three control algorithms to keep the concentration in the ECS at the target value. We set the flow rate from the drug port with three patterns described below:

1. Keep the flow rate at the constant value of 5.5 μL/min (Constant; same as Figure 8).
2. Change flow rate depending on the measured ECS concentration. The flow rate was calculated as 5.5 μL/min × (target concentration)/(measured concentration). The flow rate increased as the measured concentration decreased. (Proportional feedback).
3. Calculate the appropriate flow rate by the digital twin of the microreactor. The detail is described in Section 2.6. The flow rate was calculated after each measurement. (Digital twin).

**Figure 9:**
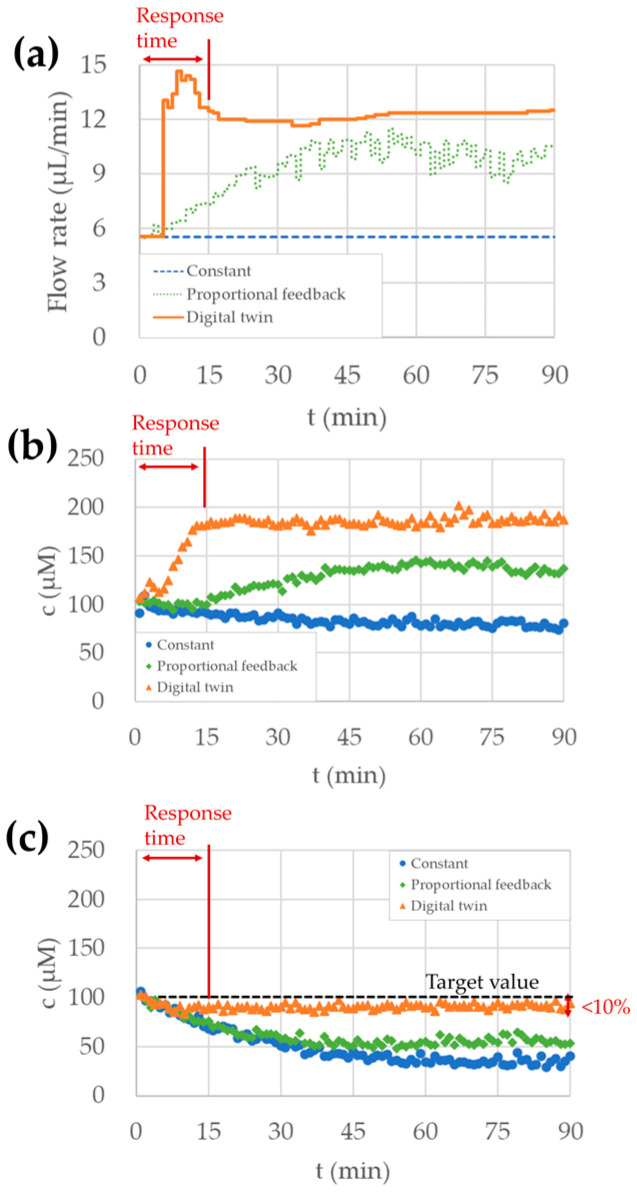
Flow rate and concentration in the hollow fiber microreactor measured with three different control methods. The drug destruction rate in the ECS was set to −2.6 μM/min in all control methods. (**a**) The flow rate of the drug port in the ICS controlled by three different methods: constant rate, proportional feedback, and digital twin. (**b**) Concentration measured by the sensor in the ICS. (**c**) Concentration measured by the sensor in the ECS.

Figure 9 shows the flow rate from the drug port and the concentrations measured using different control algorithms. Figure 9a shows the flow rate from the drug port, which indicates that the flow rate was changed during the evaluation in both control methods with the proportional feedback and the digital twin.

Figure 9b shows the concentration in the ICS, which indicates that the concentration was enhanced in the control methods with proportional feedback and numerical model. In both control methods with the proportional feedback and digital twin, a larger amount of sodium chloride was supplied, which led to higher concentrations in the ICS. On the other hand, the concentration in the ICS decreased due to destruction in the ECS with a constant flow rate.

Figure 9c shows the concentration in the ECS, which indicates that the concentration became closest to the target value in the control methods with the digital twin. With the proportional feedback method, sodium chloride was not supplied enough to keep the concentration at the target value. The concentration in the ECS had average values of 92 μM in the control methods with the digital twin (75~90 min). This result indicates that we can control the drug concentration in the ECS within an error of 10% using the digital twin. Moreover, the control method with the digital twin stabilized the drug concentration in both the ICS and ECS within a response time of 15 min, which is quicker than the other control methods and quick enough in HFIM operation, which takes several hours or days. With a high-density bacteria culture where drug destruction is not negligible, the digital twin of the hollow fiber microreactor is effective in keeping drug concentration in the ECS to the target value.

## 4. Conclusions

In this paper, we developed a hollow fiber microreactor applicable to the HFIM, creating an integrated cartridge with HFIM functions to reduce the consumption of culture medium and drugs. The whole ECS volume in the microreactor is 180 μL, which is less than 1/10 of that in conventional hollow fiber cartridges.

In order to ensure drug diffusion through hollow fibers and accurately control the drug concentration in the ECS, we constructed a numerical model that calculates drug advection and diffusion through hollow fibers as a digital twin of the microreactor. By combining a concentration measurement and the numerical model by EnKF, the diffusion flux through hollow fibers was estimated within 30 min, which was quick enough in HFIM operation, which takes several hours or days. This helps ensure drug administration to the ECS, which cannot be achieved in the conventional HFIM.

We simulated drug destruction by bacteria to check the accuracy of the drug concentration in the ECS. The concentration became less than half of the target value with conventional HFIM control that supplies solutions with target concentration continuously. On the other hand, we can control the drug concentration in the ECS within an error of 10% using the digital twin. What is more, the control method with the digital twin stabilized the drug concentration in both the ICS and the ECS within a response time of 15 min. By estimating unknown properties of the microreactor, such as the diffusion flux through hollow fibers and the drug destruction rate, our control method with digital twin can control the concentration in the ECS more precisely compared with other methods. Moreover, the response of the concentration is quicker than that of other methods. For hollow fiber microreactors applicable to HFIM, the utilization of the digital twin concept is effective in both the estimation of the microreactor’s properties and precise control of the microreactor.

## Figures and Tables

**Table 1:** Constants in Equations (8)–(10).

Constants	Values
$E_{p}+(EI^{*})$	950 μM
$k_{d}$	$0.3\;s^{-1}$
$k_{f}$	$0.01\;{\mu M}^{-1}s^{-1}$
$k_{3}$	$0.005\;s^{-1}$

**Table 2:** Flow rate settings for pumps in the drug administration process.

Pumps	Flow Rate
ICS diluent port	10.0 μL/min
ICS drug port	5.5 μL/min (First 30 min) 1.1 μL/min (Otherwise)
ICS circulation	600.0 μL/min
ICS elimination port	150.0 μL/min
ECS circulation	150.0 μL/min
ECS elimination port	0.0 μL/min

**Table 3:** Flow rate settings for pumps in simulating drug destruction.

Pumps	Flow Rate
ICS diluent port	50.0 μL/min
ICS drug port	5.5 μL/min
ICS circulation	600.0 μL/min
ICS elimination port	150.0 μL/min
ECS circulation	150.0 μL/min
ECS elimination port	Corresponds to 2.6 μM/min destruction
ECS diluent port	Corresponds to 2.6 μM/min destruction

## Data Availability

The original contributions presented in this study are included in the article/Supplementary Materials. Further inquiries can be directed at the corresponding author.

## Supplementary Materials

The following supporting information can be downloaded at https://www.mdpi.com/article/10.3390/mi15121517/s1; Table S1: Measured/calculated values in Figure 7; Table S2: Measured values in Figure 8; Table S3: Measured/calculated values in Figure 9.
