# Supplementary material for: Hollow Fiber Microreactor Combined with Digital Twin to Optimize the Antimicrobial Evaluation Process
Source: Micromachines (Basel). 2024 Dec 20;15(12):1517. doi: 10.3390/mi15121517 (PMC11677925; doi:10.3390/mi15121517)
Supplement: Supplementary file 1 [file micromachines-15-01517-s001.zip › micromachines-3344791-supplementary.pdf]

Hollow Fiber Microreactor Combined with Digital Twin to Optimize Antimicrobial Evaluation Process

Kazuhiro Noda, Toshihiro Kasama, Marie Shinohara, Masakaze Hamada, Yukiko T. Matsunaga, Madoka Takai, Yoshikazu Ishii and Ryo Miyake

Table S1. Measured/ calculated values in Fig. 7

t(min)	c(mM) Sensor1	c(mM) Sensor2	q (μL/min)	t(min)	c(mM) Sensor1	c(mM) Sensor2	q (μL/min)	t(min)	c(mM) Sensor1	c(mM) Sensor2	q (μL/min)
1.00	0.05	0.05	12.48	31.00	1.76	0.56	9.37	61.00	1.26	0.96	9.44
2.00	0.09	0.06	16.17	32.00	1.75	0.58	9.73	62.00	1.26	0.96	9.36
3.00	0.17	0.06	19.79	33.00	1.73	0.61	10.48	63.00	1.25	0.96	9.31
4.00	0.26	0.06	24.94	34.00	1.69	0.63	10.98	64.00	1.25	0.97	9.30
5.00	0.35	0.06	26.63	35.00	1.67	0.65	11.05	65.00	1.24	0.97	9.25
6.00	0.43	0.07	26.59	36.00	1.65	0.67	10.88	66.00	1.24	0.98	9.10
7.00	0.49	0.08	23.38	37.00	1.62	0.69	10.77	67.00	1.23	0.98	9.08
8.00	0.56	0.09	21.31	38.00	1.60	0.71	10.91	68.00	1.22	0.99	9.00
9.00	0.63	0.10	17.92	39.00	1.58	0.73	10.91	69.00	1.22	0.99	9.07
10.00	0.68	0.12	15.61	40.00	1.55	0.74	10.75	70.00	1.21	1.00	9.09
11.00	0.75	0.13	13.38	41.00	1.54	0.76	10.76	71.00	1.21	1.00	9.07
12.00	0.84	0.14	12.64	42.00	1.52	0.77	10.36	72.00	1.20	1.00	9.09
13.00	0.91	0.16	12.16	43.00	1.51	0.79	10.33	73.00	1.20	1.01	8.93
14.00	0.96	0.18	11.48	44.00	1.49	0.80	10.14	74.00	1.19	1.02	8.95
15.00	1.02	0.20	10.82	45.00	1.47	0.82	10.27	75.00	1.19	1.02	8.93
16.00	1.07	0.21	10.58	46.00	1.45	0.82	10.04	76.00	1.18	1.03	8.66
17.00	1.12	0.23	10.19	47.00	1.43	0.84	10.16	77.00	1.18	1.03	8.97
18.00	1.17	0.26	9.97	48.00	1.42	0.85	10.20	78.00	1.17	1.03	8.78
19.00	1.22	0.27	9.00	49.00	1.40	0.86	10.17	79.00	1.17	1.03	8.83
20.00	1.26	0.29	9.43	50.00	1.39	0.87	10.01	80.00	1.17	1.03	8.79
21.00	1.32	0.31	9.01	51.00	1.38	0.88	10.04	81.00	1.15	1.04	8.62
22.00	1.41	0.34	8.90	52.00	1.36	0.89	10.17	82.00	1.15	1.04	8.69
23.00	1.46	0.36	9.14	53.00	1.35	0.90	10.10	83.00	1.15	1.04	8.65
24.00	1.50	0.39	9.27	54.00	1.34	0.91	9.89	84.00	1.15	1.04	8.84
25.00	1.54	0.41	9.37	55.00	1.33	0.92	9.78	85.00	1.15	1.05	8.83
26.00	1.57	0.43	9.43	56.00	1.31	0.92	9.65	86.00	1.14	1.05	8.74
27.00	1.60	0.46	9.30	57.00	1.31	0.93	9.60	87.00	1.11	1.05	9.16
28.00	1.63	0.48	8.80	58.00	1.30	0.94	9.62	88.00	1.10	1.05	9.26
29.00	1.66	0.51	9.27	59.00	1.29	0.95	9.58	89.00	1.11	1.05	9.27
30.00	1.69	0.53	9.76	60.00	1.28	0.95	9.65	90.00	1.11	1.06	9.24

Table S2. Measured values in Fig. 8

t(min)	c(mM)_Sensor1	c(mM)_Sensor2	t(min)	c(mM)_Sensor1	c(mM)_Sensor2	t(min)	c(mM)_Sensor1	c(mM)_Sensor2
1.00	91.41	106.15	31.00	87.38	48.68	61.00	79.62	33.52
2.00	109.49	96.91	32.00	85.92	53.15	62.00	77.15	40.34
3.00	98.31	96.41	33.00	86.04	49.86	63.00	85.11	35.74
4.00	96.33	93.97	34.00	79.98	51.33	64.00	77.10	35.64
5.00	93.44	89.58	35.00	85.79	41.75	65.00	80.17	36.83
6.00	93.48	92.74	36.00	83.14	44.13	66.00	78.56	31.61
7.00	94.05	84.86	37.00	79.88	46.04	67.00	76.81	34.70
8.00	89.90	88.81	38.00	81.93	49.21	68.00	81.58	33.89
9.00	96.15	82.07	39.00	82.71	40.61	69.00	77.92	32.64
10.00	92.15	79.68	40.00	81.93	40.47	70.00	80.22	31.80
11.00	93.50	77.37	41.00	77.00	43.07	71.00	82.15	32.31
12.00	92.09	74.34	42.00	84.11	42.77	72.00	82.08	34.83
13.00	91.20	80.76	43.00	84.46	42.18	73.00	77.78	32.27
14.00	92.41	70.98	44.00	81.46	38.27	74.00	76.84	34.29
15.00	90.74	67.71	45.00	79.82	44.87	75.00	75.59	39.94
16.00	89.35	66.77	46.00	86.06	41.68	76.00	80.93	32.00
17.00	90.64	68.32	47.00	81.47	41.68	77.00	83.23	31.17
18.00	89.26	70.82	48.00	82.51	36.14	78.00	82.53	31.73
19.00	86.07	68.67	49.00	80.10	38.80	79.00	82.86	44.12
20.00	91.23	67.95	50.00	78.45	41.14	80.00	82.29	36.03

21.00	91.48	58.28	51.00	78.37	40.36	81.00	83.35	36.70
22.00	86.95	60.79	52.00	81.89	42.44	82.00	76.67	31.10
23.00	86.76	63.29	53.00	79.51	35.30	83.00	77.15	32.11
24.00	87.83	56.17	54.00	84.97	35.85	84.00	77.25	40.05
25.00	89.08	57.85	55.00	80.57	33.82	85.00	76.32	29.40
26.00	83.24	61.18	56.00	77.16	32.45	86.00	80.40	34.23
27.00	84.31	57.85	57.00	83.87	37.23	87.00	76.72	37.50
28.00	87.96	57.47	58.00	87.32	33.52	88.00	75.37	30.14
29.00	86.86	53.93	59.00	80.56	42.60	89.00	74.15	35.13
30.00	91.24	51.17	60.00	77.76	34.46	90.00	80.76	40.72

Table S3. Measured/ calculated values in Fig. 9

t(min)	Flow rate (µL/min)			c(mM)_ICS			c(mM)_ECS		
	Constant	Proportional feedback	Digital twin	Constant	Proportional feedback	Digital twin	Constant	Proportional feedback	Digital twin
1.00	5.55	5.55	5.55	91.41	102.99	107.10	106.15	101.49	102.42
2.00	5.55	5.47	5.55	109.49	101.43	111.86	96.91	97.31	100.70
3.00	5.55	5.70	5.55	98.31	101.61	122.94	96.41	90.09	96.47
4.00	5.55	6.16	5.55	96.33	103.71	117.98	93.97	97.35	93.11
5.00	5.55	5.70	5.55	93.44	101.18	112.52	89.58	92.59	94.14
6.00	5.55	5.99	13.06	93.48	99.22	116.15	92.74	92.89	94.00
7.00	5.55	5.98	12.65	94.05	99.46	124.80	84.86	87.48	89.01
8.00	5.55	6.34	13.39	89.90	94.49	139.66	88.81	86.33	85.80
9.00	5.55	6.43	14.64	96.15	101.75	146.81	82.07	86.12	91.39
10.00	5.55	6.44	14.18	92.15	100.67	159.85	79.68	78.33	83.65
11.00	5.55	7.09	14.43	93.50	100.26	164.40	77.37	80.80	89.78
12.00	5.55	6.87	14.19	92.09	95.52	176.79	74.34	76.10	90.96
13.00	5.55	7.29	13.45	91.20	102.23	181.14	80.76	75.93	92.49
14.00	5.55	7.31	12.68	92.41	99.74	181.39	70.98	75.11	87.67
15.00	5.55	7.39	12.68	90.74	99.48	182.09	67.71	75.55	90.28
16.00	5.55	7.35	12.45	89.35	107.48	185.24	66.77	70.15	88.85
17.00	5.55	7.91	12.36	90.64	105.29	182.97	68.32	71.22	92.87
18.00	5.55	7.79	12.00	89.26	108.87	184.66	70.82	69.02	86.23
19.00	5.55	8.04	12.00	86.07	109.08	184.31	68.67	66.38	92.76
20.00	5.55	8.36	12.00	91.23	110.19	185.72	67.95	66.09	86.76
21.00	5.55	8.40	12.00	91.48	118.30	189.23	58.28	62.36	90.65
22.00	5.55	8.90	12.00	86.95	113.60	188.93	60.79	61.70	89.93
23.00	5.55	9.00	12.00	86.76	114.29	189.16	63.29	59.79	91.59
24.00	5.55	9.28	11.90	87.83	116.85	186.85	56.17	59.23	88.05
25.00	5.55	9.37	11.90	89.08	119.68	184.94	57.85	67.63	90.76
26.00	5.55	8.21	11.90	83.24	118.51	184.54	61.18	62.18	87.77
27.00	5.55	8.93	11.90	84.31	118.78	183.50	57.85	62.49	86.65
28.00	5.55	8.88	11.90	87.96	120.41	187.03	57.47	62.75	91.75
29.00	5.55	8.84	11.90	86.86	121.71	181.75	53.93	61.78	89.54
30.00	5.55	8.98	11.90	91.24	120.22	181.11	51.17	57.79	90.26
31.00	5.55	9.60	11.90	87.38	113.67	184.32	48.68	58.82	94.94
32.00	5.55	9.44	11.90	85.92	126.97	182.47	53.15	55.28	88.76
33.00	5.55	10.04	11.90	86.04	127.98	185.84	49.86	55.72	94.82
34.00	5.55	9.96	11.67	79.98	122.13	189.21	51.33	63.95	89.44
35.00	5.55	8.68	11.67	85.79	129.98	185.48	41.75	53.23	88.04
36.00	5.55	10.43	11.67	83.14	126.28	182.48	44.13	53.64	89.01
37.00	5.55	10.35	11.67	79.88	129.34	176.42	46.04	51.22	85.74
38.00	5.55	10.84	11.77	81.93	124.75	182.38	49.21	51.60	91.20
39.00	5.55	10.76	11.77	82.71	129.09	187.20	40.61	59.94	86.45
40.00	5.55	9.26	12.00	81.93	137.71	181.76	40.47	54.45	91.41
41.00	5.55	10.19	12.00	77.00	132.71	187.33	43.07	51.05	90.48
42.00	5.55	10.87	12.00	84.11	136.53	187.69	42.77	55.59	97.24
43.00	5.55	9.98	12.00	84.46	135.92	187.72	42.18	53.88	90.45
44.00	5.55	10.30	12.00	81.46	134.91	184.79	38.27	51.17	89.34

45.00	5.55	10.85	12.00	79.82	135.10	182.75	44.87	51.07	88.57
46.00	5.55	10.87	12.00	86.06	134.48	183.72	41.68	56.50	91.66
47.00	5.55	9.82	12.00	81.47	136.54	183.15	41.68	51.78	91.51
48.00	5.55	10.72	12.08	82.51	134.32	181.41	36.14	51.46	91.95
49.00	5.55	10.79	12.08	80.10	139.93	183.65	38.80	49.19	87.15
50.00	5.55	11.28	12.18	78.45	137.49	183.70	41.14	55.26	88.94
51.00	5.55	10.04	12.18	78.37	138.67	192.33	40.36	51.08	90.37
52.00	5.55	10.87	12.27	81.89	140.75	186.44	42.44	57.56	93.01
53.00	5.55	9.64	12.27	79.51	137.86	185.84	35.30	55.88	91.91
54.00	5.55	9.93	12.27	84.97	136.53	183.93	35.85	48.37	87.73
55.00	5.55	11.47	12.36	80.57	136.38	182.15	33.82	51.89	90.26
56.00	5.55	10.70	12.36	77.16	141.94	181.33	32.45	49.59	94.85
57.00	5.55	11.19	12.36	83.87	139.94	183.41	37.23	52.95	91.64
58.00	5.55	10.48	12.36	87.32	143.03	188.68	33.52	50.41	92.49
59.00	5.55	11.01	12.36	80.56	145.95	180.41	42.60	53.84	92.59
60.00	5.55	10.31	12.36	77.76	142.44	183.56	34.46	55.28	90.14
61.00	5.55	10.04	12.36	79.62	144.30	183.72	33.52	51.72	90.47
62.00	5.55	10.73	12.36	77.15	144.56	191.71	40.34	51.97	86.55
63.00	5.55	10.68	12.36	85.11	140.58	183.96	35.74	61.85	95.49
64.00	5.55	8.97	12.36	77.10	141.58	179.43	35.64	54.20	93.84
65.00	5.55	10.24	12.36	80.17	140.12	191.05	36.83	59.52	90.99
66.00	5.55	9.33	12.36	78.56	142.86	184.86	31.61	51.31	92.60
67.00	5.55	10.82	12.36	76.81	138.08	188.85	34.70	58.22	91.01
68.00	5.55	9.53	12.36	81.58	139.53	201.82	33.89	55.42	92.11
69.00	5.55	10.01	12.36	77.92	135.26	192.11	32.64	52.55	90.37
70.00	5.55	10.56	12.36	80.22	141.63	197.72	31.80	51.30	95.53
71.00	5.55	10.82	12.36	82.15	144.23	184.14	32.31	55.52	90.56
72.00	5.55	10.00	12.36	82.08	140.14	184.25	34.83	52.43	90.74
73.00	5.55	10.59	12.36	77.78	138.77	190.67	32.27	59.22	87.32
74.00	5.55	9.37	12.36	76.84	145.11	192.46	34.29	56.30	91.64
75.00	5.55	9.86	12.36	75.59	138.53	183.45	39.94	53.82	94.79
76.00	5.55	10.31	12.36	80.93	141.52	183.77	32.00	63.02	91.50
77.00	5.55	8.81	12.36	83.23	143.72	186.89	31.17	61.67	89.03
78.00	5.55	9.00	12.36	82.53	138.29	185.59	31.73	54.49	92.48
79.00	5.55	10.19	12.36	82.86	132.65	190.88	44.12	65.01	93.50
80.00	5.55	8.54	12.36	82.29	133.88	186.79	36.03	61.64	91.91
81.00	5.55	9.00	12.36	83.35	133.37	185.83	36.70	56.94	95.45
82.00	5.55	9.75	12.36	76.67	130.20	186.22	31.10	54.15	93.64
83.00	5.55	10.25	12.36	77.15	134.68	191.48	32.11	58.54	91.79
84.00	5.55	9.48	12.36	77.25	138.71	187.06	40.05	57.71	90.67
85.00	5.55	9.62	12.45	76.32	131.97	186.39	29.40	53.37	92.76
86.00	5.55	10.40	12.45	80.40	136.80	193.16	34.23	55.47	90.16
87.00	5.55	10.01	12.45	76.72	133.11	185.15	37.50	56.22	96.29
88.00	5.55	9.87	12.45	75.37	132.09	185.74	30.14	52.71	88.49
89.00	5.55	10.53	12.45	74.15	131.55	191.22	35.13	52.64	87.80
90.00	5.55	10.54	12.53	80.76	137.00	187.06	40.72	53.48	93.93
